# Biomimetic Nanoparticles Coated with Bacterial Outer Membrane Vesicles as a New-Generation Platform for Biomedical Applications

**DOI:** 10.3390/pharmaceutics13111887

**Published:** 2021-11-07

**Authors:** Atanu Naskar, Hyejin Cho, Sohee Lee, Kwang-sun Kim

**Affiliations:** Department of Chemistry and Chemistry Institute for Functional Materials, Pusan National University, Busan 46241, Korea; atanunaskar@pusan.ac.kr (A.N.); chj9512@pusan.ac.kr (H.C.); kin5497170@pusan.ac.kr (S.L.)

**Keywords:** bacterial outer membrane vesicles (OMVs), membrane coating, biomimetic nanoparticles, biomedical applications

## Abstract

The biomedical field is currently reaping the benefits of research on biomimetic nanoparticles (NPs), which are synthetic nanoparticles fabricated with natural cellular materials for nature-inspired biomedical applications. These camouflage NPs are capable of retaining not only the physiochemical properties of synthetic nanoparticles but also the original biological functions of the cellular materials. Accordingly, NPs coated with cell-derived membrane components have achieved remarkable growth as prospective biomedical materials. Particularly, bacterial outer membrane vesicle (OMV), which is a cell membrane coating material for NPs, is regarded as an important molecule that can be employed in several biomedical applications, including immune response activation, cancer therapeutics, and treatment for bacterial infections with photothermal activity. The currently available cell membrane-coated NPs are summarized in this review. Furthermore, the general features of bacterial OMVs and several multifunctional NPs that could serve as inner core materials in the coating strategy are presented, and several methods that can be used to prepare OMV-coated NPs (OMV-NPs) and their characterization are highlighted. Finally, some perspectives of OMV-NPs in various biomedical applications for future potential breakthrough are discussed. This in-depth review, which includes potential challenges, will encourage researchers to fabricate innovative and improvised, new-generation biomimetic materials through future biomedical applications.

## 1. Introduction

Researchers are constantly attempting and implementing new innovative approaches to overcome various caveats in efficient biomedical applications. Cell membrane-coated nanoparticle (NP) is one of these innovative approaches where synthetic NPs are coated with cell-derived membranes to fabricate a core–shell structure that can be used in various biomedical applications [1,2]. Erythrocytes or red blood cells (RBC), leukocytes or white blood cells (WBC), platelets, cancer cell membrane, and bacterial cell membrane have been used at various depths to coat NPs and to generate a camouflaged NP for various biomedical applications [3,4]. The obvious advantage of this cloaked NP system is the tunability of the physicochemical properties of the synthetic NPs, while the bio-interfacial properties and functions of natural bio-membranes remain intact in the cloaked set-up, enabling NPs to freely enter the cell system.

Among the various types of cell membranes, bacterial outer membrane vesicles (OMVs) have demonstrated a great potential in biomedical applications; however, they are fairly new in the biomimetic design combined with NPs [5]. OMVs are secreted by all bacterial species, including Gram-negative and -positive bacteria, and have a particle size of 20 to 300 nm in nature [6]. OMVs have been demonstrated to be enriched in bioactive proteins, toxins, virulence factors, and immunogenic materials to stimulate bacteria-host interactions, which are important for vaccine development [5,7]. Moreover, the non-replicative nature of OMVs is regarded as generally safe relative to that of their parental bacteria and alters their biological roles in unpredictable ways via different environmental stresses [8]. Therefore, OMVs could have effective uses in various in vivo biomedical applications.

The properties of OMVs can be further exploited to coat NPs when fabricating biomimetic NPs [9,10]. The potential to use NPs for biomedical application is huge, as several NP-based studies have been conducted for biomedical applications owing to the unique and tunable physiochemical properties of NPs [11,12]. Nonetheless, some issues remain a challenge and must be addressed, such as toxicity to ensure that the potential of NPs are fully utilized. For in vivo applications, NPs may absorb cellular components after entry into the body, which might result in changes to these NPs, to ensure their long-term performance [13]. Moreover, in most cases, the surface of the NPs needs to be properly functionalized to achieve more diversified biomedical applications. Different strategies have been utilized successfully to functionalize NPs for excellent biomedical application [14,15]. In this regard, the coating of NPs with OMVs as ghost NPs has shown great promise for biomedical application [16,17,18,19,20]. Coating NPs with bacterial OMVs (OMV-NPs) endows the NPs with the biological functions of OMVs, enabling this cloaked arrangement to yield more desired applications. These OMV-NPs not only preserve the tunable physicochemical properties of synthetic NPs but also enhance the biological functions of OMVs. Moreover, coating of NPs with OMVs is achieved through a facile process without the use of any organic solvents, thereby reducing some of the limitation of traditional surface modifications and offering good biocompatibility.

In this review, we first introduce the classification of cell membranes and their coated NPs, which are broadly utilized in current biomedical applications. Thereafter, we describe OMV-NPs and their biomedical applications; this includes a detailed description of the nature, composition, and functions of OMVs and different nanocores used for the core part of OMV coating. Current strategies to fabricate OMV-NPs and their characterization to demonstrate the formulation of biomimetic NPs are also presented. Finally, the biomedical applications of OMV-NPs and the prospects and challenges of this novel formation of new-generation materials are described.

## 2. Classification of Cell Membrane-Coated NPs

**Figure 1:**
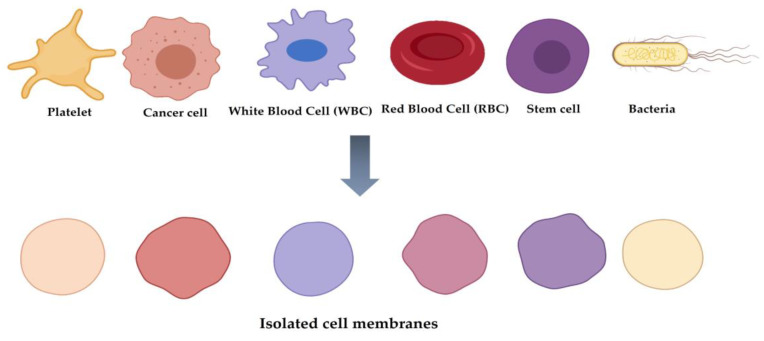
Schematic of different isolated cell membranes.

The progress of cell membrane-coated NPs and their applications in the biomedical field has been demonstrated by the fabrication of various types of cell membranes and their coating of NPs, which is depicted in Figure 1.

The first in this category is **erythrocyte** or **red blood cell** (RBC) membrane-coated NPs [21]. RBC membranes have been employed to coat NPs for biomedical applications owing to their properties such as high circulation time, immune evasion, and mimicking cell glycocalyx to prevent serum protein adsorption and resistance to complement reactions [3]. Owing to the abovementioned features, various particles, such as gold NPs, mesoporous silica NPs, and poly(lactic-co-glycolic acid) (PLGA) NPs, have been coated with RBC membranes for different biomedical applications.

Similarly, **leukocyte** or **white blood cell** (WBC) membranes [22] have been used to coat NPs for biomedical research applications owing to the ability of WBC membranes to easily cross biological barriers, their long blood circulation time, and their ability to identify the diseased or inflamed regions. Mesoporous silica nanocapsules, composite capsules of sodium alginate, Au nanoshells, and chitosan have been utilized as inner cores with the WBC membrane coating for various biomedical applications such as long blood circulation time, photothermal anticancer applications, and others [2,3].

Similar to the RBCs and WBCs, **platelets** are essential components in mammalian blood cells and understandably have attracted remarkable attention from researchers for the fabrication of membrane-coated NPs; this is because of their hemostasis and immune response properties [23]. Based on these favorable properties, NPs coated with the membrane components of platelets have been successfully used [2,3] for site-specific delivery, cancer therapy, and enhanced magnetic resonance imaging.

The assessments related to RBCs, WBCs, and platelets for biomimetic NPs are reasonably logical owing to their favorable physiochemical properties for biomedical applications. However, the unorthodox research carried out with NPs coated with **cancer cell membrane** for biomedical application was quite surprising, as the membrane was part of a cancer cell. Astonishingly, NPs coated with the cancer cell membrane showed good results when evaluated for biomedical application [24]. The effectiveness of these NPs was aided by several unique properties of the cancer cell membrane, including limitless replicative potential, immune tolerance, resistance to cell death, long circulation time, and homologous binding capabilities. PLGA NPs, Fe_3_O_4_ NPs, and Au nanocages have also been coated with cancer cell membranes for different biomedical purposes [25].

NPs have also been coated with the membrane components of **mesenchymal stem cells** (MSCs). As MSCs show different favorable features, such as inhibition of inflammation and site-specific targeting of damaged tissues [26], membrane components of MSCs are useful for fabricating core NPs to achieve greater biocompatibility in nature. Additionally, membranes of MSCs can be harvested from various tissues, enabling various modification opportunities for future applications. For example, iron oxide (MRI applications) and gelatin NPs (tumor-pH-sensitive drug release behavior and high drug-loading capacity) were successfully coated with the membranes of MSCs for different biomedical purposes [2].

Dual membrane-coated NPs (i.e., **hybrid** NP systems), in which erythrocyte and platelet membranes are fused together to coat NPs, have been fabricated and evaluated for in vivo application based on their long circulation time and has the potential to overcome the limitations of current nanoparticle-based therapeutic and imaging platforms [27].

A recent development in this category is bacterial cell membrane-coated NPs [7]. The initiation of cellular and humoral immune responses along with bacterial survival in anaerobic environments has prompted researchers to use bacterial cell membrane-coated NPs for biomedical applications [16,17,18,19,20]. Despite the fewer research studies on bacterial membrane-coated NPs than those on mammalian-derived membrane-coated NPs, the former has shown remarkable promise for further application-oriented studies in terms of following aspects. First, OMVs could be a novel and potentially ideal source of cellular membrane for coating NPs in applications requiring the activation of pathogen-associated innate and adaptive immune responses [28,29] as well as the prevention of inflammation caused by commensal bacteria resided in the gut [30]. This promising application comes from the fact that OMVs include a high content of immunogenic proteins and adjuvants that can be used to modulate the immune system of target hosts [31]. Second, OMVs have the potential to target specific cell types in an active and efficient manner [32]. For instance, OMVs from *Staphylococcus aureus* (*S. aureus*) accumulated more readily in organs with high bacterial burdens, such as the kidney, spleen, lungs, and heart. Third, the facile modification and high production of OMVs by general molecular biological approaches could be achieved in less time and at a lower cost. Overall, OMVs with above innate abilities have the potential to improve therapeutic outcomes by overcoming limitations of NPs such as low cellular uptake by target cells, low immunogenicity, cytotoxicity, biocorona formation on their surface, non-selective targeting, and enhanced clearance rates. These properties of OMVs in the coating with NPs may be valuable to researchers in a variety of biomedical applications.

PLGA NPs [19] and Au NPs [16] have already been used as core components in bacterial cell membrane coatings for biomedical applications. The bacterial membrane coating approach offers a great potential to solve currently challenging biomedical issues. Moreover, the use of bacterial membrane offers a safe, suitable, and effective surface engineering strategy, which would not be achieved if bacteria themselves were directly used. In terms of safety, the bacterial membrane coating is biocompatible in living organisms, as demonstrated by different anticancer oriented studies [18]. Finally, the unlimited potential of bacterial membrane coatings can only be achieved through further optimization and modifications. The coating, modification, and applications of NPs coated with bacterial cell membrane are thoroughly discussed in a later section.

## 3. Bacterial OMVs and Their Features in Biomedical Applications

OMVs are nano-sized and non-replicative spherical particles with a diameter ranging from 20 to 300 nm that are released by both pathogenic and non-pathogenic Gram-negative and -positive bacteria [6], from their outer membrane (OM) into the extracellular milieu [33]. Bacterial OMVs are well known to affect diverse biological processes, including virulence, horizontal gene transfer, export of cellular metabolites, phage infection, and cell-to-cell communications. Additionally, OMVs display remarkable potential in various biomedical applications. Current OMVs are generally known to be produced through controlled blebbing of the OM of bacteria [34]; however, molecular mechanisms underlying OMV biogenesis from bacterial species remain elusive at present [34]. The currently knowledge on the major components and functions of OMVs, which have prompted researchers to use OMVs to coat NPs in biomedical applications, is discussed in later sections.

### 3.1. Components of OMVs

Bacteria-generated OMVs contain a variety of parent bacteria-derived bioactive periplasmic and cytoplasmic proteins, nucleic acids (DNA and RNA), lipids, and virulence factors, and these materials contribute to the maintenance of bacterial ecosystem and bacteria–host communications [34]. The sorting and structure of individual bioactive components and their loading as cargo control the composition, size, and distribution of OMVs, thereby controlling their specificity in counteracting the modes associated with target bacteria and host responses. The major biomedical applications that facilitate the use of OMVs in coating nanomaterials include vaccines, adjuvants, cancer immunotherapy agents, drug delivery vehicles, and antibacterial agents [35]. In these applications, the components of the OMVs aid the host or communicating bacteria in expressing more effective biomedical outcomes through novel cellular physiology modulating routes compared with previously used molecules. In the following sections, we discuss the major components of OMVs.

#### 3.1.1. Proteins

An abundance of OM proteins (OMPs; OmpA, OmpC, and OmpF), periplasmic proteins (AcrA and alkaline phosphatase), misfolded proteins, and virulence factors involved in the adherence and invasion of host tissues was discovered in OMVs [33]. After OMVs were purified via ultracentrifugation, over 3500 proteins belonging to various functional categories were characterized using MS-based proteomic profiling methods. However, as evidenced by global proteomic analysis, OMVs lack the components of inner cell membrane and the cytoplasm [36], suggesting that the vesiculation is a directed process rather than a random event. As the current general biogenesis of OMVs is known to originate from blebbing and turgor pressure [37], enveloped components of OM proteins and periplasmic proteins are major components in OMVs. In recent years, the collection of high-throughput proteomic data of OMVs from pathogens and commensal bacteria has revealed new functional proteins that could be utilized in biomedical applications. Proteomic profiling of OMVs, for instance, revealed the presence of membrane-bound proteins such as transporters, receptors, signaling molecules, protein channels, and proteins from various cellular origins [38]. On the other hand, separate proteomic profiling analysis data vary among different data sets, making it impossible to rely on proteomic data from multiple individual investigations. To overcome these challenges, globally coordinated standard proteomic data has been complied and is currently available to public. For instance, ProteomeXchange [39] was open to public for this purpose, and pathogen-derived OMV proteomic data [40] is progressively accumulated in the database. This database also includes species comparison research results [41]. With accumulated information, new modulatory components in OMVs in certain biological processes are identified. In addition, potential biomarkers linked to the gut microbial ecosystem in the microbiota, pathogenicity, and disease progression are identified. These proteins, which are linked to certain biological pathways, are applied to create effective platforms for biomedical applications. Therefore, integrated proteomics under a variety of physiological conditions are necessary to employ OMV as an important platform for designing broadly applicable biomimetic materials.

#### 3.1.2. Nucleic Acids

Bacterial OMVs carry luminal and surface-associated DNA, RNA, plasmid, phage DNA, and chromosomal DNA. According to a recent report, the presence of DNA in OMV was confirmed via analysis following DNase treatment; the resistance of luminal DNA persisted even after the treatment process [42]. Furthermore, several different forms of luminal DNA have been reported from *Escherichia coli (E. coli)*, *Neisseria gonorrhoeae (N. gonorrhoeae)*, *Pseudomonas aeruginosa (P. aeruginosa)* and *Haemophilus influenza (H. influenza)* [43]. Moreover, genomic DNA in OMV was discovered in commensal bacterium, *Enterobacter cloacae* [38]. Small non-coding RNAs (sRNAs) from bacteria, ranging in length from 50 to 400 nucleotides, have been found in OMVs in recent years from bacteria and were also identified in OMVs. For instance, Ghosal and colleagues discovered the existence of sRNAs in *E. coli* MG1655-derived OMVs [44], which contain 15 and 40 nucleotides in size RNA fragments with diverse functions. In addition, sRNAs from *V. cholerae* [45], uropathogenic *E. coli* strain 536, and *P. aeruginosa* were found within OMVs [46,47]. Furthermore, periodontal pathogens such as *Aggregatibacter actinomycetemcomitans*, *Porphyromonas gingivalis*, and *Treponema denticola*, have been shown to secrete sRNAs in OMVs [48]. More interestingly, sRNAs packed inside OMVs derived from *S. typhimurium* were not degraded by ribonucleases (RNases) [49]. As shown above, bacteria seem to utilize OMVs as cargo for functional sRNAs to escape from the degradation by RNases and to be used in other biological functions in associated host or bacteria. Functional sRNAs derived in OMVs in bacteria have been identified by discovery-based high-throughput RNA-sequencing studies, as shown in a previous report [47], even though their functions and the reason of presence have not been fully characterized.

Nucleic acids involved in the bacterial communications and host–pathogen interactions were identified. For instance, RNA-containing OMVs play a major role in bacteria-host interactions [45]. Moreover, chromosomal DNA for encoding proteins implicated in virulence, stress responses, antibiotic resistance, and metabolism in *P. aeruginosa*, found in both the internal compartments of *P. aeruginosa*-derived OMVs and host eukaryotic cells, presumably by host–OMV interaction [50]. However, mechanistic investigations of how foreign DNA is integrated into communicating cells have yet to be completed. Furthermore, the role of sRNAs in OMVs in intercellular communications was suggested. For instance, sRNA52320, a fragment of a tRNA^Met^, was transferred to human airway cells, resulting a reduction of interleukin-8 (IL-8) levels by its targeted regulation to mRNA encoding MAP-kinase [47]. In addition, sRNAs to target host mRNA function was differentially packaged in *H. pyroli*-derived OMVs and attenuated IL-8 secretion in human cells to evade the host immune response [51]. The complete understanding of the OMV-mediated communication route through nucleic acids between bacteria and bacteria, or bacteria and host is required for the determination of biological functions. Therefore, future studies on the transfer mechanism are required. This knowledge could also be used to build OMV/nucleic acid-based vaccines, particularly for bacterial infections.

#### 3.1.3. Lipids

Lipids play a vital role in the structure of bacterial OMVs. It is postulated that certain lipid components have a role in OMV biogenesis based on the enriched and excluded OMV components. Lipopolysaccharide (LPS) and phospholipids (PLs) are the most common lipid components of OMV produced from Gram-negative bacteria. While PLs constitute the inner sheet of the OM, LPS is exclusively located on the outersurface of the OM. Many studies have revealed that the PL content of Gram-negative bacteria is varied [52]. The PL content of *E. coli* OMVs mainly consists of phosphatidylethanolamine (PE), phosphatidylglycerol (PG), and lysophosphatidylethanolamine [53], whereas PG and PE [54], or PG and stearic acid [55] are major lipid components in *N. meningitidis* or *P. aeruginosa* OMVs, respectively. LPS-deficient *Myxobacterium Sorangium cellulosum* So ce56 contains three primary lipid classes of OM including sphingolipid (SL), a lipid class characterized by a long-chain amino alcohol backbone, glycerol ether lipids (GEL), and ornithine-containing lipids (OL). Group A *Streptococcus* (GAS)-derived OMVs contain more than 85 individual glycerol lipids (GL), both anionic and cationic PLs, and anionic PGs [56]. Cardiolipin (CL) is the major lipid component in Gram-positive *H. pylori* OMVs [56]. Gram-negative bacteria generally contain both neutral (O-LPS) and negatively charged (A-LPS) O antigen residues in OM. Among them, only A-LPS has been discovered in Gram-negative bacteria-derived OMVs [33]. The fact that gingipains, which are highly abundant proteins in *P. gingivalis*, were abolished in A-LPS mutant strain highlighted the relevance of A-LPS in OMV packaging.

The delivery of *P. gingivalis* SLs to host cells by OMVs was recently demonstrated to alter the host inflammatory response during cellular infection [57]. Since OMVs from *Spingomonas paucimobilis* contain SL, a lipid class that plays important structural and signaling roles in eukaryotes, this bacterium would be a smart producer to create a platform candidate that takes advantage of host–bacterial interaction [58].

It is also well-recognized that the differences in lipid content have an impact on the rigidity of OMVs. Even-numbered carbon chain fatty acids (accounting for more than 80% of the fatty acids contained in the OMVs) were substantially concentrated in OMVs of the Antarctic bacterium *Pseudomonas syringae (P. syringae)* [59]. As a result, an enhanced membrane flexibility for OMV-related applications was proposed [60]. Otherwise, in comparison with the OM, studies of the fatty acid content of *P. aeruginosa* OMVs indicated that these OMVs were enriched with longer and more saturated fatty acids, suggesting that the more rigid portions of the OM are prone to producing OMVs [53].

The asymmetric lipid distribution in OMVs may help to potentiate cargo loading. The occurrence of nanoscale lipid domains in bacterial membranes, such as flotillins, was reported in *B. subtilis* [61].

Overall, the lipid composition in bacteria is critical for constituting compositions in OMVs, implying that changing bacterial OM could be a potential way to modulate OMV compositions and functions. Therefore, high-throughput lipidomics with the mechanistic investigations for various OMVs with different lipid components, and functions or applications are required to understand the relationship between lipid components and OMV functions.

#### 3.1.4. Virulence Factors

Several studies have shown that OMVs directly communicate between bacteria and the host by delivering toxins and enable bacterial virulence in host cells [62] to benefit pathogenic bacterial species. For instance, EstA, a bacterial virulence factor derived from the OMVs of *P. aeruginosa*, induced nitric oxide and pro-inflammatory cytokines during macrophage interaction with host cells [36]. Owing to such virulence factors, host cells were mis-regulated by OMV-released proteins associated with proteolysis, ion transport, and ion binding [36]. *H. pylori-*derived OMVs presented Lewis antigens on their surface and could induce the activation of the host immune system [63]. In this case, the OMVs directly bound to anti-Lewis antibodies in serum to decrease the self-defense ability of host cells, thereby playing an important role in *H. pylori* pathogenesis. As described above, OMVs play a critical role in various biomedical applications, as they deliver various toxins and activate the host defense system. This suggests that OMVs could also be applied to various nano-platforms for different biomedical issues.

### 3.2. Functions of OMVs

OMVs can export different cellular components along with NPs [16,17,18,19,20]. These specific cargo molecules enable functions that mediate bacteria–bacteria communications, immunomodulatory host–bacteria communications, and cell detoxification.

#### 3.2.1. Bacteria–Bacteria Communications

OMVs can interact with one another as well as with other microorganisms in their environment and with the host. In addition, OMVs have special communication systems under changing or challenging environmental conditions, including quorum sensing (QS), biofilm formation, nutrient acquisition, antibiotic resistance, and competition with or defense against other microbes [64]. For instance, *Paracoccus denitrificans* and *Vibrio harveyi* OMVs use quorum sensing to interact with other bacteria and host cells under activated quinolone signals (PQSs), including C16-HSL and CAI-1 [64]. In communities, OMVs share biofilm matrix to improve the overall function of bacteria for nutrient uptake and survival. In *P. aeruginosa*, planktonic and biofilm-derived OMVs have quantitative and qualitative differences and possess proteolytic activity and antibiotic-binding abilities, suggesting that they are engaged in some of the activities assigned to biofilms [65]. In addition, OMVs from antibiotic resistance strains have shown that they transfer antibiotics-resistant genes and proteins to susceptible strains [35,60]. For example, carbapenem-resistant *A. baumannii* OMVs were packed with carbapenem resistance-related genes and could undertake the horizontal transfer to carbapenem-susceptible *A. baumannii* [35]. Additionally, OMVs generated from *E. coli* contained colistin, which degrades antimicrobial peptides such as melittin [66]. *Moraxella catarrhalis* and *S. aureus* OMVs carried β-lactamase and evaded bacteria from β-lactam antibiotics [67].

The principal OMV-mediated process through DNA is thought to be horizontal gene transfer. However, only a few studies have looked into OMVs’ ability to gene transfer [68]. There are three steps in this process: releasing DNA from donor bacteria encapsulated with OMVs, merging OMVs with the OM of the recipient bacteria, and migrating genetic materials into the cytoplasm of the recipient bacteria. The instances and factors were extensively described [68], and this review does not go over them in detail. However, further studies are needed to fully understand the mechanisms for loading and attachment of OMVs to recipient bacteria as well as factors that influence the processes. Furthermore, the new OMV mechanism in bacterial communications should be investigated in species with varying lipid compositions.

#### 3.2.2. Immunomodulatory Host–Bacteria Communications

OMVs are highly effective in modulating innate immune responses in the host without direct host cell–bacterial interactions since they contain many immune-reactive proteins originating from their parent bacterium [69]. In terms of structure and biological activity, bacteria-derived OMVs are similar to mammalian cell-derived EVs (exosomes). To induce the immunomodulatory activity, OMVs should alter the integrity of the epithelial barrier to be required for maintaining homeostasis in mammalian cells linked to inflammatory, allergic, and metabolic diseases [70]. Furthermore, an interaction between the gut microbiome and the intestinal epithelium is critical for the barrier integrity. For instance, OMVs from probiotic *E. coli* Nissle 1917 and commensal ECOR63 were able to enhance barrier function [71].

Besides the interaction of OMVs with the host epithelial cells, OMVs directly affected numerous immune cells, resulting in either activation or suppression of immune responses in the host, which was observed in OMVs from both Gram-negative and -positive bacterial species. *H. pyroli* OMVs have been found within infected gastric mucosa of *H. pyroli*-infected patients who suffered from gastric disease [72]. *Salmonella typhimurium (S. typhimurium)* OMVs promote flagellin-mediated caspase-1 activation and IL-1β secretion in an endocytosis-dependent manner [69]. OMVs from *H. pyroli*, *Neisseria*, *Pseudomonas*, *Campylobacter*, and *Vibrio* cause non-phagocytic epithelial cells to secrete interleukin-8 (IL-8), a chemokine generated by a number of tissues and blood cells [73,74]. Gram-positive bacteria including commensal *Lactobacillus* (L.) *plantarum* and *L. sakei* OMVs protect against pathogen infection by stimulating host defense genes [64]. A certain sRNA carried in *A. actinomycetemcomitans* OMVs activated the pro-inflammatory cytokine TNF-α via the TLR-8 and NF-κB signaling pathways in human macrophages [75].

The importance of the OMV in the suppression of immune responses have also been reported. For instance, differentially packaged sRNAs in *H. pyroli*-derived OMVs attenuated IL-8 secretion in human cells to evade the host immune response [51]. Furthermore, OMVs could protect Gram-negative bacteria against host defense peptides (HDPs), part of the innate immune system, and could comprise first-line defense against invading pathogens [76].

All of this evidence suggests that OMVs play a pivotal role in both immune responses and host cell homeostasis. Combining bacterial OMVs with NPs results in the induction of host cell immune response for anticancer activity [18]. Therefore, new OMV-NP platforms need to be developed. This will be useful for the immune-related clinical therapies, as it will prevent the immune clearance seen with other single-use medications in clinics.

#### 3.2.3. Cell Detoxification: Toxins, Adhesions, and Virulence Factors

Many pathogenic bacteria produce OMVs that contain toxins, degradative enzymes, virulence factors, and pro-inflammatory chemicals. Such components can also be used as a tool to promote their contact with host cells [77]. For instance, *Actinobacillus pleuropneumoniae* OMVs induce hemolysis and cytolysis by activating the Apx toxin (Shiga toxin). However, *S. typhimurium* possesses a specific apparatus, termed type III secretion system (T3SS), that secretes over 40 virulence components involved in the interaction with host cells [78]. Although the roles of OMV-associated virulence factors have not been fully characterized, it is clear that the survival of *Salmonella* species inside the host is reduced when these factors are absent [77]. These OMV-induced toxins are typically protease-resistant despite being found on the exterior surface of vesicles.

## 4. Inner Core NPs

NPs are becoming a potential therapy for treating diseases such as bacterial infections caused by resistance to currently available antibiotics; such resistance is one of the most serious issues threatening human health worldwide [11,79,80]. Several properties of NPs, including large surface-area-to volume ratio, small size, modification of target ligand, and cost effectiveness, serve as advantages for their use as alternative agents to resolve current antibiotic-related challenges [81]. To date, only a few NPs have been approved by the US Food and Drug Administration (FDA) owing to their toxicity. Potential toxicity caused by the chemical materials used to synthesize NPs has been previously reported; this toxicity should be extensively ameliorated to enable the future clinical use of NPs [82,83]. To overcome this issue, cell biomimetic technology, involving the use of bacterial membrane through coating systems, has recently received increasing attention. Coating NPs with cell membranes enhances the biocompatibility of NPs, thereby enabling researchers to utilize both the chemical and physical properties of synthetic NPs as well as the biological properties of host cells. Inner core NPs also play a crucial role in the production of cell membrane-coated NPs, as they are effective at delivering the information of NPs to target cells [84]. Thus, they should be selected based on the requirement of cargo delivery.

### 4.1. Organic NPs

Liposomes, polymer constructions, and micelles are examples of organic nanomaterials that display biocompatibility, biodegradability, and low toxicity. These advantages are widely utilized in different applications, such as imaging, drug delivery systems, and vaccine platforms [85]. The advantages of high processability, good flexibility, and less toxicity enable researchers to use various organic NPs for biomedical applications. Different NPs used for cell membrane coating are discussed in this section.

#### 4.1.1. PLGA NPs

PLGA, which is already approved by the FDA, is generally applied as a platform for anticancer therapy because it has a high capacity to transport cancer drugs and displays stabilization in physiological buffer [86]. Its efficacious use for biomedical applications can be attributed to hydrolysis, which results in the generation of lactic acid and glycolic acid, the two monomers that are endogenous and easily metabolized via the Krebs cycle in the body and produce minimal systemic toxicity for biomedical applications [87]. For example, PLGA was coated on the B16-F10 cancer membrane to improve the cellular uptake and immunological responses, thereby serving as an antigen delivery system [88]. Similarly, platelet membrane-coated PLGA NPs containing rapamycin, a potent immunosuppressant and anti-inflammation activator, targets atherosclerotic plaque owing to their strong platelet binding affinity and displays good stability for 7 days [89]. RBCs have also been used to coat PLGA NPs, which provide a unique strategy for cargo delivery by avoiding attack by the immune system [90].

#### 4.1.2. Liposomes

Liposomes with at least one lipid bilayer have a spherical form and are commonly used as nanocarriers in drug delivery applications, as they have been approved by the FDA for clinical usage [91]. The importance of liposomes has been demonstrated, as more than 18 liposomal drugs have been approved by the FDA to treat different conditions such as cancer, infectious disease, pain, and others [92]. Their unique biphasic nature enables them to deliver both hydrophilic as well as lipophilic drugs, which is one of the reasons for their excessive use in biomedical applications. RBC membrane-coated liposome containing amphotericin B (RBC-LIP-Amb), an antifungal drug used to treatment of fungal infections, inhibits the growth of the pathogenic fungi *Candida albicans* and prolongs retention time of the drug when infected [93].

#### 4.1.3. Other NPs

Bovine serum albumin (BSA) is another NP used in inner core materials due to its high availability and inexpensive cost [94]. In the presence of reactive groups such as thiol and amine, it is extremely water-soluble and flexible, allowing it to interact with other compounds such as drugs and inorganic materials for use in drug delivery systems by allowing drugs to be transported in the body through regulating drug encapsulation. For example, RBC-coated particles comprising BSA with 1,2-diaminocyclohexane-platinum (II) (DACHPt) and indocyanine green (ICT) target tumors and are controlled with appropriate targeting peptides [95].

### 4.2. Inorganic NPs

Inorganic NPs (e.g., gold NPs, mesoporous silica NPs, and metal oxides) exhibit distinct characteristics compared with organic NPs, such as a smaller material size, high stability, enhanced permeability, and high drug capacity. Moreover, their tunable physiochemical properties and large surface-to-volume ratio support their use by researchers as core components in various cell membrane coatings in different biomedical applications [85].

#### 4.2.1. Gold NPs

Gold NPs (Au NPs) have been successfully evaluated to demonstrate their non-toxicity, enhancement of immunogenic activity, antioxidant activity, and gene delivery owing to their unique physical and chemical properties [96]. Additionally, Au NPs are well known as excellent antibacterial agents among NPs [97]. Therefore, predictably, researchers use Au NPs in the coating with cell membranes for various biomedical applications. For example, *E. coli* OMV-coated Au NPs with a size of 30 nm were found to induce a significant production of interferon gamma (INF-γ) and interleukin-17 (IL-17), both of which are indicators of T-cell-mediated immune response. Furthermore, Au NPs were found to promote the high activation and maturation of dendritic cells in lymph nodes as well as to generate antibody responses with higher avidity [16]. Therefore, they could be employed in tumor vaccination or treatment. Gold nanocages (Au NCs) with tunable near-infrared (NIR) absorption and strong photothermal ability have been used to effectively treat cancer cells. Instead of using polyethylene glycol (PEG) as a stabilizer of gold, RBC membranes are used to protect the degradation of Au NCs in blood and improve tumor uptake [98].

#### 4.2.2. Mesoporous Silica NPs

Mesoporous silica nanoparticles (MSNPs) have been widely used in biomedical applications because of their controllable particle size, high specific surface area, low toxicity, hemocompatibility, and ease of surface modification. Specifically, their ability to regulate drug release via modification with particular response to various stimuli, such as pH, chemical agents, redox agents, and lights, makes them suitable drug delivery systems that can release appropriate targets [99]. Doxorubicin (DOX)-loaded MSNPs were coated with OMVs from *E. coli* and were subsequently integrated with neutrophils, which are essential components of immune cells with intrinsic chemotaxis capability [100]. Consequently, it enables the uptake of MSNPs into neutrophils without loss of cellular activity and motility. Similarly, cancer cell membranes [101] and RBC membranes [102] were used to coat MSNPs for different biomedical applications.

#### 4.2.3. Iron Oxide NPs

Iron oxide NPs are universal magnetic nanomaterials for clinical MRI [103]. Such NPs also enable surface alteration, thereby providing long circulation for hyperthermia therapeutic systems. With respect to cell membrane coating, DOX loaded with iron oxide NPs with cancer cell membrane (DNP@CCCM) exhibits excellent in vitro targeting, in vitro cytotoxicity, stability, and hemocompatibility [104].

## 5. Potential Advantages of OMV-NPs

There are a number of instances of cell membrane-coated NPs and their applications in biomedical challenges. The utilization of these camouflage combinations can be attributed to various reasons, including selective permeability and cellular communication properties of cell membrane-coated NPs. In biomedical applications, OMV-NPs have the same potential as native OMVs.

**Selective permeability** is one of the required properties in biomedical application. OMV can be a great ally for this purpose due to its good natural properties to overcome issues from NPs. NPs are commonly employed in preparing nanocarriers for targeted drug delivery. For instance, liposomes, mesoporous silica NPs (MSNPs) and iron oxide NPs have been successfully utilized as drug nanocarriers [105]. However, there are still challenges to overcome the limitations of their applications. Cell penetration [106] can be readily remedied using OMV. Moreover, OMV-NPs have a higher biocompatibility than individual NPs because the body treats the cell membrane as its own. Due to the aqueous environment in the interior of the vesicle, cell membranes are potential nanocarriers of hydrophilic reagents. Synthetic nanomaterials, on the other hand, are good drug nanocarriers with hydrophobic agents and a high loading capacity. Additionally, it can co-deliver drugs with varying hydrophilicity. However, their low biocompatibility prevents them from being used in biomedical application. In this regard, combining OMV with synthetic nanomaterials can be employed synergistically for drug delivery, as OMV-NP combinations not only have the potential for excellent nanocarriers but also exhibit excellent drug loading capacity with selective permeability. Furthermore, OMV-NP combinations allow for the nanomaterial to easily penetrate the bacterial cell since the bacteria consider OMV to be a part of their own body. This feature may result in high antibacterial activity in situations when NP penetration is problematic. Biocompatibility is another property that can be used for various in vivo applications or wound healing activity.

**Cellular communication** is another potential benefit of OMV-NP combinations. Bacterial cells do not attack or block OMV owing to their self-recognition properties. Various studies have used cellular communication property of cell-derived membrane-coated NPs in various biomedical applications, such as prolonged blood circulation, targeted therapy, immunity, and others [107]. First, when combined with synthetic nanomaterials, bacterial OMVs contain some immunogenic antigens that can elicit strong antibacterial immune responses. More in-depth discussion is given in latter section (Section 8.3. Antibacterial vaccine). As a result, the immunity application of OMV-NPs has already been demonstrated using their cellular communication features [16]. Second, apart from immunization, OMV-NPs have the ability to prolong blood circulation, as demonstrated by different cell-derived membrane-coated nanomaterials [108]. Nanomaterials have long circulation period, which aids systemic and targeted delivery [109]. However, nanomaterials need some appropriate modifications before they can be applied in biomedical applications as the immune system does not allow the nanomaterials to enter the body system since they are not true biological products. Hence, cell-derived membrane-coated camouflaged nanomaterials are not affected by such consequences as immunity systems do not attack the cell membrane. Therefore, OMV-NPs have the potential to be used in a variety of biomedical applications, where prolonged circulation period is required by hiding from body immune system. Third, another potential application for OMV-NPs is targeted therapy, which requires the use of cellular communication properties. The targeted therapy generally requires the decoration of ligand onto NPs in order to target specific cells. The synthesis of such ligand-based nanomaterials is challenging, despite the fact that this approach is highly selective. In this regard, cell membrane-coated nanomaterials can be utilized for targeted therapy because the cell membrane has the ability to target the original cells. For this goal, stem cell or leukocyte-derived membranes have already been used. As a result of its inherent cell membrane features, the OMV may have the same potential characteristics for targeted therapy as counterparts from eukaryotic cells.

## 6. Strategies to Fabricate OMV-NPs

**Figure 2:**
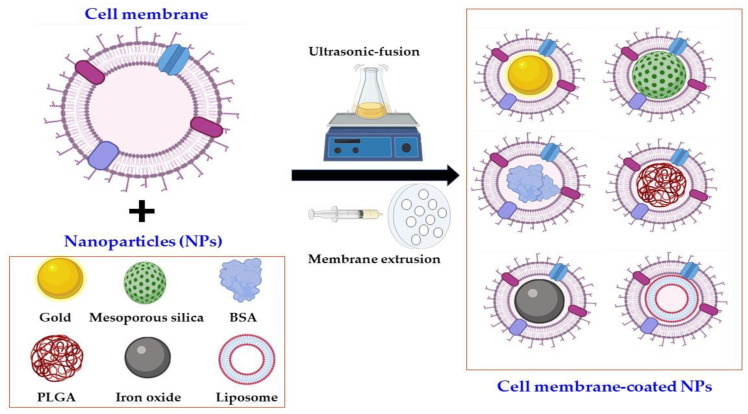
Schematic representation of different cell membrane coatings of NPs with ultrasonic fusion of the membrane extrusion process.

The fabrication of OMV-NPs (Figure 2) mainly involves three steps: preparation of OMVs, inner core production, and the coating process. These steps are discussed in detail below.

### 6.1. Preparation and Characterization of OMVs

As described in Section 3.1, OMVs contain a cell-derived phospholipid bilayer, nucleic acids, membrane proteins, and lipopolysaccharides [6,33]. OMVs have been used in many therapeutic applications, but often, their release is insignificant, resulting in low yields from bacterial cultures. In addition, as reported by various studies, different culture conditions or stresses are known to produce heterogenous OMVs of different compositions even from the same bacteria. As a result, the production of homogeneous OMVs with specific sizes or components is a remarkable challenge [110]. In the preparation ion of OMVs, currently, there is no single standardized technique available. Instead, conventional techniques, ultracentrifugation (UC) and precipitation using polymeric compounds, are generally used to isolate OMVs. Since there is a concern that each technique may alter the properties of isolated OMVs, it is still challenging to develop new reliable methods to yield OMVs with desired purity and quantity. Since OMV preparation with a desired amount is one of the main parts of OMV-NP fabrication, here, we discuss induction methods of OMVs, generally used and newly developed OMV preparation methods, a newly developed preparation method and their considerations for downstream applications, and the molecular detoxification of OMVs in preparing OMV-NP combinations.

#### 6.1.1. Induction Methods

One of the main limitations in OMV isolation and purifications is to obtain sufficient quantity of nano-sized vesicles without scale-up the process. Several methods have been presented to increase the release of OMVs. **First,** OMVs can be induced by disrupting the membrane with either physical (sonication) or chemical treatment such as extracting agent (ethlenediaminetetraacetic acid, EDTA) or sublethal concentration of antibiotics [111]. **Second,** OMVs can be induced by genetic modifications. There are many reports that OMV production is affected by individual gene modifications. As a well-known example, the Tol-Pal system has often been a target for creation of hypervesiculating mutants [112,113]. Another example is the chaperone gene knockout to increase the presence of misfolded proteins, which results in increases in the OMV formation, as shown for a *degP* mutant of *E. coli* [6]. **Third,** the control of OMV-associated protein expression is known to affect hypervesiculation, for instance, the cellular perturbation of the ratio between OmpC and OmpA proteins by MicA sRNA in *E. coli* hyperproduced OMV [114]. Although the above cases induce the production of OMVs, one major concern for the use of such OMVs in biomedical applications is whether the desired activity is retained by the modifications, since the inducing steps could alter the size, proteolytic or thermal stability, or composition of OMVs. In this regard, MicA-derived OMVs could be a good one to be used in the application of vaccine development since the OMV showed an immune responsive and protective role against *S. typhimurium* [115]. Therefore, the number of studies on the identification of inducing conditions or regulators associated with the hypervesiculation of OMV on the global scale without damaging the activity and their utilization in preparing bioengineered OMVs will increase.

#### 6.1.2. Preparation of OMVs

UC was first used in 1966 by Knox et al. [115] to prepare OMVs from Gram-negative bacteria. Since then, it has become a mainstay for the preparation of OMVs. In the first step, bacteria are cultured in an appropriate fluid medium for a specific time. Thereafter, the bacteria cells and contaminants are removed by centrifugation and the supernatant is collected following filtration using 0.22 and 0.45 μm filters. Finally, the supernatant is pelleted by UC, ranging from 40,000 up to 175,000× *g*, depending on the bacterial species studied for a certain time, which is critical for yields of OMVs but not specified in general, to obtain OMVs, with the soluble proteins remaining in the supernatant. The optimization of the cultural medium, centrifugal speed, and time could potentially produce more uniform OMVs through the UC process. Sequential density gradient centrifugation can also be applied to further improve the purity and quality of OMVs [5]. Several authors have achieved successful results for producing OMVs from *E. coli*, *P. aeruginosa*, *S. typhimurium*, *S. aureus*, *B. subtilis*, and *S. pneumonia* with uniform morphology and homogeneous size through UC.

The precipitation method is another viable approach to extract OMVs [5]. Herein, some saturated salt is used in the solution where the supernatant of the bacterial cells and debris have been kept with UC. OMVs can be precipitated successfully after this process, as the salt solution disrupts the stability of the protein solution and the proteins aggregate together, enabling easier centrifugation and their separation.

Notably, the abovementioned processes are rarely used alone for the collection of OMVs as both have limitations. First, UC requires a complex operative procedure, the time required to prepare OMVs is long, and the cost of the machines is high; these caveats limit the use of UC and, thus, the progress of potential researchers. In addition, UC alone may leave contaminants in the isolated OMV fraction. To overcome this issue, sucrose density gradient UC results in the purest fraction of OMVs [116]. Furthermore, OMV isolated by UC enhanced the isolation of larger sized vesicles (>100 nm) [117]. Second, a key and difficult point of precipitation method is the preparation of the salt solution with proper concentrations and saturations as well as the addition of homogenized salt with an adjusted slow rotation speed to avoid the formation of overconcentrated formed crystal. Furthermore, OMVs should be further dialyzed to remove the soluble salt before further use. The whole process is also complex, and the proteins on the OMVs are threatened by the concentrated proteases released from bacteria during centrifugation. Furthermore, the addition of protease inhibitors into the filtered culture media before the concentration step increases the undesired contents, resulting in a low yield of OMVs.

The dual-cyclic tangential flow filtration (dcTFF) system [118], which has been successfully used to prepare nanosized extracellular vesicles (EVs) from B16BL6 cell media, is another recently developed approach that could be used to prepare OMVs. This system is made up of two membranes with different pore sizes of 30 and 200 nm that are connected to a peristaltic pump that keeps a continuous circulation stream flowing to the membrane. In comparison with TFF, this system not only reduces clogging issues but also improves the isolation efficiency such as recovery rate and yield. For instance, the yield of EVs obtained with the dcTFF chip was 4.5 times higher than that with ExoQuick, a precipitation approach kit produced by System Biosciences (SBI). Furthermore, when compared with ExoQuick and ultracentrifuged EVs, the dcTFF chip produced more uniform-sized EVs. Therefore, this technique could be utilized to isolate size-based OMVs from bacterial cultures with a high yield in a single step.

Owing to the complications associated with the above complicated procedures, long duration, and high cost, upgrading the abovementioned techniques or finding suitable alternatives to these procedures is required to extend OMV-related investigations in the biomedical field. The first isolation system, which is commercially available for bacterial OMVs by SBI, “ExoBacteria™ OMV isolation Kit”, was introduced on the market to purify OMVs from *E. coli* and other Gram-negative bacterial species. This system is a precipitation-free, ion-exchange chromatography system containing a capture resin and gravity column for specifically capture of OMVs from a bacterial culture medium. This kit delivers OMVs < 1 h and yields UC-based purity. Although the kit is for Gram-negative, it can be used for OMV preparation for Gram-positive bacteria such as *Micrococcus*, *Streptococcus*, and *Lactobacillus* species although optimization is still required. In addition, lipid- or metabolite-based kits for bacterial species are also promising platforms since such components in bacteria vary among species.

#### 6.1.3. Molecular Detoxification of OMVs

As described in the above sections, the application of OMV in biomedicines has received the greatest attention until now. For use in the therapy of humans, however, the detoxification of these nanoparticles is necessary, especially for the case of Gram-negative bacteria containing LPS, which consists of lipid A (endotoxin) and a distal polysaccharide (O-antigen) required. Early research towards detoxification used detergent extraction to reduce the LPS content [119,120] since it is the primary toxic component of OMVs. However, detergent extraction is laborious and cost intensive and reduces the adjuvant activity, resulting in serious problems in vaccine development. To overcome this issue, a genetic modification of OMV-producing bacteria to alter the physical properties of the LPS during their growth has emerged. For instance, the *E. coli* [121], *N. meningitidis* [120], and *Bordetella pertussis* [122] strains have been engineered to alter the toxicity. Furthermore, the lipid A structure can be remodeled by supplementing the bacteria with exogenous genes that encode LPS-modifying enzymes. For instance, *H. pylori* Hp0021, an enzyme that removes the 1-phosphate from lipid A, resulted in the biosynthesis of monophosphorylated lipid A instead of the natural diphosphorylated form [123]. This type of lipid A is an FDA-approved adjuvant [124] and could be used for the engineered OMV preparations in biomedical applications, such as influenza vaccine development [125]. Recently, many commensal bacteria originated OMVs are used in the vaccine development to overcome the above issues including safety. For instance, *Bacterioides thetaiotaomicron* (Bt), a prominent member of the intestinal microbiota of all animals, was engineered in the preparation of influenza A virus-derived vaccine [126]. Overall, the toxicity of OMVs should be considered in biomedical applications of OMV-NPs.

### 6.2. Inner Core NP Production

The inner core is an important part of OMV-NP formation. Various types of NPs have been used to fabricate the inner core in cell membrane-coated formations, such as PLGA [19], liposomes [93], mesoporous silica [102], gold [16], and others. For OMV coating, PLGA [19], gold [16], hollow polydopamine (HPDA) [18], and BSA [17] have been used for different biomedical applications. The US FDA approved PLGA NPs for this purpose, owing to their excellent biocompatibility and high drug-loading capacity [86]. Liposomes are another type of NPs that have entered many clinical trials for this purpose [91]. The use of inorganic NPs as inner core nanocarriers is also increasing owing to their low cost, easy synthesis, and controllable physiochemical properties (size; shape surface composition; and optical, electrical, and magnetic features).

### 6.3. Coating or Fusion Process

The final step in the formation of OMV-NPs is the coating or fusion process with OMVs and inner core NPs. In this step, the inner core NPs must be coated with OMVs or fused in OMVs to form a biomimetic NP. Currently, two methods (**membrane extrusion** and **ultrasonic fusion**) are frequently used to coat core NPs with OMVs or any cell membrane. For cell **membrane extrusion** (Figure 2), the Avanti mini extruder is used to extrude both OMVs and inner core NPs several times through a nanoscale polycarbonate porous membrane [16,17]. Mechanical forces were the reason behind this successful coating process. BSA and Au NPs have been successfully extruded through this process where mechanical forces are used to coat the NPs with OMVs [16,17]. Although the process is extremely effective for producing OMV-NPs, it is difficult to use in large-scale production. In the **ultrasonic fusion** process, inner core NPs and OMVs are co-incubated and sonicated to generate OMV-NPs [9,19], which seems to overcome the production-scale issue. However, the lack of uniformity and different sizes of the resultant NPs are the main drawbacks of this process. Recently, a microfluidic electroporation technique that is efficient and reliable has been utilized for cell membrane-coated NP preparation [127]. Although this process has yet to be utilized for the preparation of OMV-NPs, it will be in the near future.

## 7. Characterization of OMV-NPs

Notably, the methods used to characterize or verify OMV-NPs are of utmost importance for successfully recognizing optimal preparation. The effective coating of NPs with OMVs can be evaluated through their physiochemical and biological properties.

**Figure 3:**
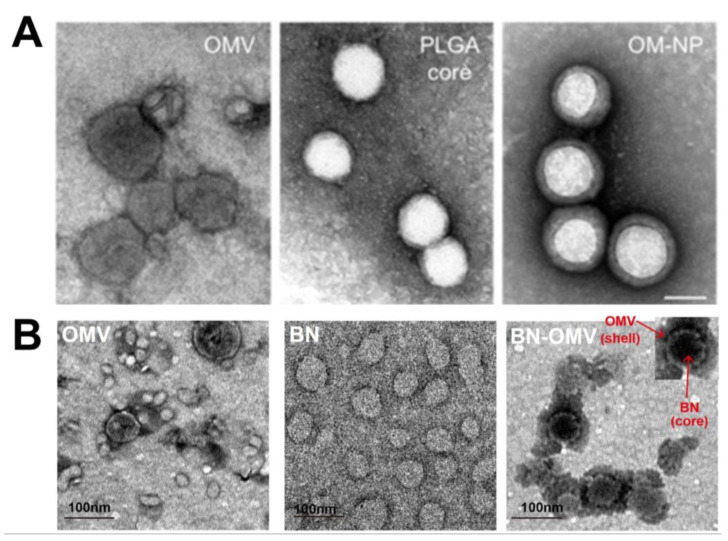
Transmission electron microscopy (TEM) images of (**A**) OMV-PLGA: OMVs, PLGA cores, and OM-NPs and (**B**) BN-OMVs: OMVs, BN, and BN-OMVs. Reproduced with permission from Reference [19] John Wiley and Sons, 2019 and Reference [17], Elsevier, 2020, respectively.

The alteration of NP size and zeta potential could be indicators of successful coating of OMVs. The change in the morphology of NPs has been compared (before and after OMV coating) using transmission electron microscopy (TEM) images. Usually, the TEM images of OMV-NP reveal a core–shell structure with increased diameter compared with that of uncoated NPs. Gao et al. [16] shared the TEM images of bacterial membrane-coated AuNPs (BM-AuNPs) with a clear spherical core–shell structure, which reflects the successful coating of gold cores in a thin shell with BM and a thickness of approximately 6 nm. Similarly, Zhang et al. [19] showed similar core–shell TEM images of OMV-PLGA samples that indicated successful coating process. In another example by Zhang et al. [19], TEM images (Figure 3A) showed that PLGA nanoparticle coated with OMV (OM-NP) maintained the spherical shape of the polymeric cores with a thin shell on the outer surface. Additionally, the work by Wu et al. [17] also showed the TEM images (Figure 3B) of BSA-OMV nanoparticles (BN-OMVs), where the core–shell structure is clearly visible. These results further confirm that TEM images can be an effective method to verify the OMV coating of NPs. Additionally, the size of OMVs should also be taken into account for successful biomedical application. For example, *H. pylori* OMVs with a size range from 20 to 100 nm preferentially entered host cells via caveolin-mediated endocytosis whereas 90 and 450 nm sized OMVs entered host epithelial cells via macropinocytosis and endocytosis [128].

Surface zeta potential is another characterization technique used to confirm the formation of OMV-NPs. Generally, the surface zeta potential of the OMV-coated NPs is increased compared with that of the core NPs [9,16,19]. For example, in a study, the zeta potential of OMV-coated PLGA NPs (−24.7 mV) was increased by almost 10 mV compared with that of pristine PLGA NPs (−34.2 mV) [9]. Similarly, Gao et al. [16] reported that the zeta potential of BM-AuNPs was −25.1 ± 0.9 mV, which is almost 13 mV higher than that of bare AuNPs (−38.6 ± 1.3 mV), indicating successful OMV coating of Au NPs. In another example, the zeta potential value increased from −40.6 ± 5.3 mV for PLGA cores to −28.8 ± 1.9 mV for OMV-PLGA NPs [19].

Dynamic light scattering (DLS) is another technique that has been used for analyzing particle size distribution to confirm the coating of NPs with OMV, as the size of the coated NPs is generally more than that of uncoated NPs. This particle size increment can be attributed to the hybrid bacterial membrane coating, which also supports characterization by TEM. In this respect, Wang et al. [18] revealed an increase in hydrodynamic size of 20 nm for OMV-NPs compared with that of the pristine NPs. Similar results were observed for OMV-PLGA [9] and BM-Au NPs [16], thereby demonstrating that particle size distribution through DLS measurement could be used to verify the OMV coating of NPs.

Despite the success of TEM, DLS, and zeta potential techniques for the confirmation of OMV-NPs, verifying the biological activities of the OMV-NPs is also necessary. Hence, different methods have been used to verify whether the coated NPs retain the hybrid cell membrane proteins. In this regard, sodium dodecyl sulfate-polyacrylamide gel electrophoresis (SDS-PAGE), protein bicinchoninic acid (BCA) assay, and Western blotting have been used to confirm the coating process and the retention of proteins. For example, using the BCA assay, Zhang et al. [19] found a markedly high protein content in OMV-PLGA NPs but not in PLGA core NPs. Similar results were also obtained for BM-Au NPs [16] and OMV-PLGA [9]. Therefore, these well-developed techniques with continuous optimizations can be successfully utilized to confirm the coating process.

## 8. Future Directions of OMV-NPs in Biomedical Applications

In the past few years, researchers have attempted to combine the advantages of natural, cell membrane-derived vesicles with those of typically effective synthetic NPs [1]. As discussed in previous sections, the structure of such biomimetic NPs mainly comprises a core–shell with a layer of cell membrane coated around the NP core. The key advantage of these biomimetic NPs is their innate ability to perform biological functions owing to their camouflage biological structure. Although OMV-coated NPs are a fairly new concept in this category, they have immense potential for biomedical applications and must be thoroughly explored. In this section, the biomedical applications of OMV-NPs are discussed.

### 8.1. Cancer Immunotherapy

**Figure 4:**
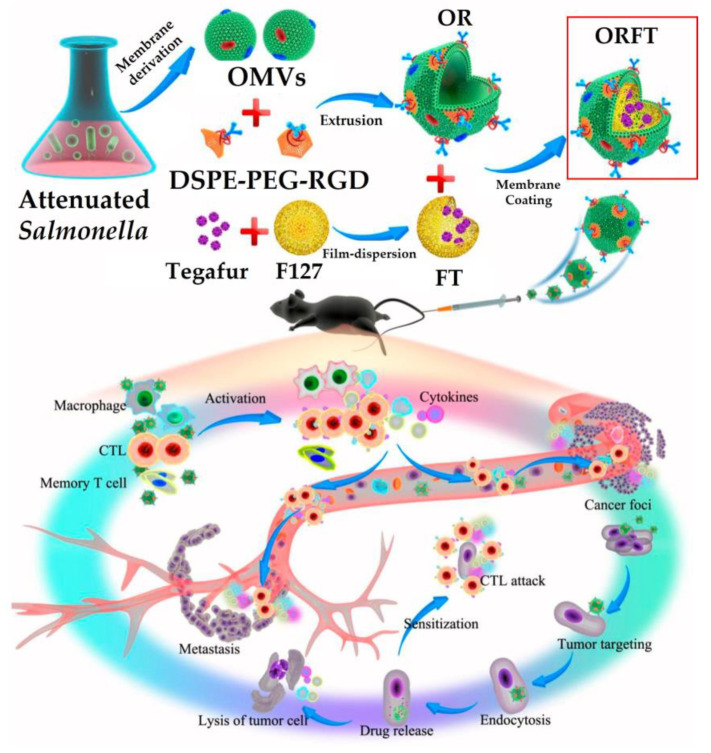
Schematic of the functionalized OMV-coated polymeric micelles for anticancer application. First, OMVs were collected from attenuated Salmonella and fused with non-fouling polyethylene glycol-PEG (DSPE-PEG) and tumor-targeting ligand Arg-Gly-Asp (RGD) through the extrusion of OMVs and DSPE-PEG-RGD to generate OMV-DSPE-PEG-RGD (OR). OR was further used to coat tegafur-loaded micelles (FT) and to obtain OR-coated F127 nanomicelles (ORFT; red box). Later, melanoma-bearing mice was intravenously injected with ORFT nanoparticles, which generate antitumoral response by OMVs and kill cancer cells directly through tegafur. Reproduced with permission from Ref. [10], copyright American Chemical Society, 2020.

The use of bacterial OMVs as immunotherapeutic tools for cancer therapy has received remarkable attention in recent years [18,20]. However, the exact mechanism of bacterial OMV-based immunotherapy remains unknown. Nonetheless, the facultative anaerobic bacteria are known to infect weakly antigenic tumors, which is believed to promote the immunogenicity of tumors and to enable cancer immunotherapeutic effects [20]. *Clostridium novyi*, *L. moncytogenes*, and *S. typhimurium* are the bacteria mainly used for this kind of immunotherapy. Chen et al. [10] fabricated a promising biomimetic nanomedicine where drug-loaded polymeric micelles were coated with bacterial OMVs to achieve effective cancer immunotherapy and metastasis prevention (Figure 4). In this synergistic approach, OMVs appeared to activate the host immune response for cancer immunotherapy, whereas polymeric micelles with the loaded drug performed both chemotherapeutic and immunomodulatory roles to kill cancer cells directly with the help of cytotoxic T lymphocytes. This camouflaged nanomedicine provided effective protective immunity against melanoma occurrence as well as tumor growth inhibition in vivo, thereby extending the survival rate of melanoma mice. In another study by Wang et al. [18], an OMV and B16-F10 cancer cell (CC) membrane, called OMV-CC hybrid membrane, was successfully coated onto hollow polydopamine (HPDA) NPs to serve as a synergistic photothermal therapy (PTT) for melanoma owing to its antitumor efficacy. These HPDA@[OMV-CC] NPs exhibited remarkable antitumor response by synergistically targeting the melanoma and stimulating dendritic cell (DC) maturation in lymph nodes to activate the immune response in vaccinated mice. This nanomedicine entirely eradicated melanoma without causing notable adverse effects.

Despite its attractiveness for biomedical applications, OMVs have received little attention in tumor therapy. The reason for this is because tumor cells use an immune escape mechanism of tumor cells in the form of IFN-γ-responsive PD-L1 expression to suppress the T cell function [129], which eventually leads to immune tolerance and limits the anti-tumor application of OMVs. As a result, new strategies, such as genetically modified OMV, might be required to address this problem. More studies are also required to see if modified OMVs with nanomaterials can be used for cancer immunotherapy. Furthermore, future research effort in this field could include the exploration of diverse chemotherapeutic agents loaded onto OMV-NPs for in vivo applications.

### 8.2. Antibacterial Activity

**Figure 5:**
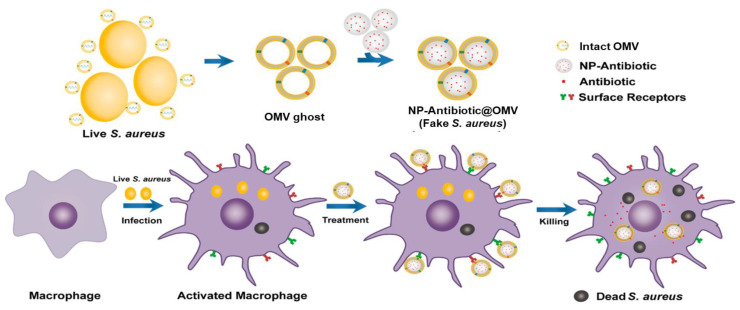
Schematic of the preparation of NP@OMV and its antibacterial activity. Reproduced with permission from Reference [9], copyright American Chemical Society, 2019.

The most intriguing feature of bacterial OMVs is their ability to be used to kill the bacteria itself. The principle is to kill the real bacterial using ghost bacteria. Traditional antibiotics fail to enter host cells, as multidrug-resistant bacteria prompt their physiological system to transform in various ways to prevent the functions of antibiotics against them. Researchers thus seek to employ NPs as antibiotic carriers [130] or use NPs as antibacterial agents [131], as some NPs have their own antibacterial properties. However, some issues, such as toxicity and free passage to enter bacterial cells, need to be carefully resolved. Accordingly, the requirement of combining OMV and NPs was established, as OMVs enable the free passage of OMV-NPs into bacteria without any blockage; this is because OMVs can be recognized by bacteria as their own and are thought to be biocompatible. In this regard, Gao et al. [9] used the FDA-approved PLGA NPs and coated the membrane of *S. aureus* OMV to actively target *S. aureus-*infected macrophages in vitro (Figure 5). The NP@OMV (NP@EV) particle as an active-targeting antibiotic carrier, with counterparts coated with PEGylated lipid bilayer (i.e., NP@Lipo; PEG = poly(ethylene glycol)), not only targeted the *S. aureus*-infected macrophage but also was found to target the major organs (kidney, lung, spleen, and heart) bearing metastatic infections in *S. aureus* bacteremia-bearing mouse models. Therefore, this NP@OMV enabled the particle to achieve an active targeting capacity both in vitro and in vivo. Consequently, NP@OMV is extremely effective when intravenously administered with preloaded antibiotics to alleviate metastatic infection in *S. aureus* bacteremia-bearing mouse model. Thus, the ever-increasing problems of antibacterial resistance can be resolved by using bacterial OM-coated NPs [7].

Similar to any other approved systems, OMV-NPs also need to encounter different challenges before it can be fully realized for antibacterial activity. Safety and immunogenicity are of prime importance for any nanocomposite to be used for in vivo antibacterial activity. Therefore, the focus needs to be directed towards developing OMV with lower amounts of attenuated immunogenic molecules using *E. coli of msbB* gene knockout [132]). Furthermore, pegylation [133] or the incorporation of anti-phagocytic CD-47 molecules [134] can also be utilized to prevent immune recognition. However, these modifications can additional complexity and need further research work for in vivo application.

### 8.3. Antibacterial Vaccine

**Figure 6:**
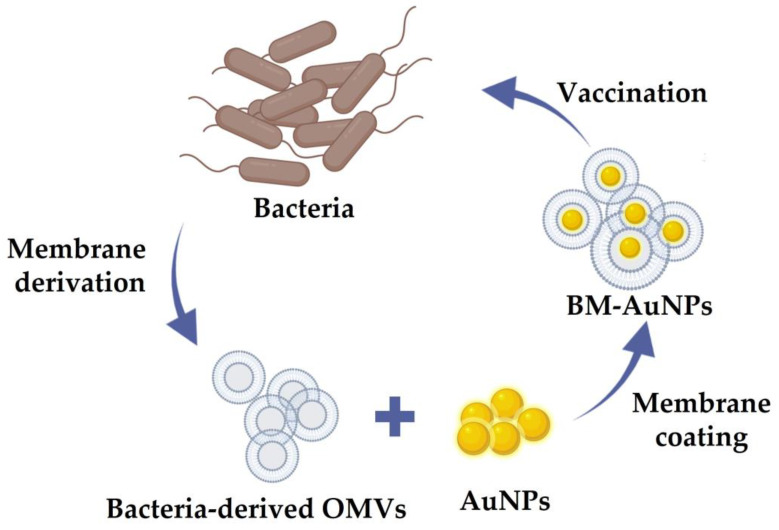
Schematic of the preparation of BM-AuNPs and their application for antibacterial vaccine. Reproduced with permission from Reference [16], copyright American Chemical Society, 2015.

Vaccines are currently a hot topic because of the COVID-19 pandemic [135] and are also an excellent way of handling bacterial infections for a long time. Vaccines are effective against various serious infections caused by pathogenic bacteria and prevent various epidemics. Despite its initial success, however, there remain some limitations in their use as an alternative to antibiotics. These limitations can be attributed to various factors, including a limited understanding of host–pathogen interactions [136] and the consequences of developing resistance of vaccination by microorganisms [137]. Another issue is the rapid emergence of bacterial drug resistance [11]. The importance of NP can be realized in this scenario as the possibility of bacteria becoming resistant against NPs much less [11]. Therefore, researchers are attempting to use the bacterial OM-coated NPs as an antibacterial vaccine to improve immune responses and to potentially enhance antimicrobial immunity [7]. Notably, bacterial membranes contain a large number of immunogenic antigens with intrinsic adjuvant properties that play a key role in promoting adaptive immune responses [6,34]. Thus, the bacterial membranes can be considered attractive vaccination materials. Meanwhile, synthetic NPs have their own tunable physicochemical properties for effective antigen presentation to immune cells. Hence, the cloaking of NPs with bacterial membrane not only preserves the unique biological characteristics of bacteria to imitate the natural antigen presentation of bacteria to the immune system but also enables the use of the physicochemical properties of the core synthetic NPs. Following these principles, Gao et al. [16] generated *E. coli* OMV-coated Au NPs (OMV-AuNPs) or bacterial membrane-coated AuNPs (BM-AuNPs) for use as antibacterial vaccines (Figure 6). The BM-AuNPs showed excellent stability in biological buffer solutions and induced rapid activation and maturation of DCs in the lymph nodes of vaccinated mice after subcutaneous administration. Additionally, these NPs generated durable antibody responses and comparatively higher avidity than OMVs alone. Furthermore, an elevated production of interferon gamma (INFγ) and interleukin-17 (IL-17) was attained through the BM-AuNPs but not interleukin-4 (IL-4). This result showed the capability of BM-AuNPs for the generation of strong Th1- and Th17-biased cell responses against the source bacteria. This work showed great potential for the generation of an effective antibacterial vaccine. In a similar study by Xu et al. [17], OMVs from carbapenem-resistant *Klebsiella pneumonia* (CRKP) were coated onto size-controlled BSA NPs through a hydrophobic interaction to fabricate a uniform and stable antibacterial vaccine. This BSA-OMV NP showed remarkably enhanced antibacterial vaccine properties. The survival rate of mice infected with a lethal dose of CRKP also increased significantly after BSA-OMV NP immunization. These bacterial membrane-coated NP combinations showed remarkable potential as cloaked nanomedicines for further antibacterial vaccine development.

For progress in the future, there is plenty of works to be completed before OMV-NPs are fully realized into a usable antibacterial vaccine. In this regard, OMV-coated NPs can be utilized with already investigated noninvasive vaccine applications, including microneedles [138] and hydrogel patches [139], for safe, rapid, and suitable vaccination. Additionally, biomaterial scaffolds can be utilized with camouflaged NPs for the recruitment of antigen presenting cells along with an environment for prolonged immune activation [140]. Likewise, OMV-NPs can be integrated into nanogels [141] to overcome the “coatability” restriction imposed by the core nanoparticle and allow for the encapsulation of immunostimulatory agents for more diversified application. Last but not least, OMV-NPs also have the potential to overcome antibiotic resistance of pathogens although detailed exploration is further needed.

### 8.4. Inhibition of Pathogen Adhesion

The inhibition of pathogen adhesion to host cells is an innovative approach to limiting bacterial infections, as the adhesion of pathogenic bacteria to host cells and tissues is the primary cause of the initiation of bacterial infections [142]. The simple and basic advantage of this anti-adhesion-based work is that it does not directly interfere with bacterial cycles for killing, unlike traditional antibiotics. Moreover, the increased likelihood of acquiring bacterial resistance is markedly lower than that with traditional antibiotics. This process also helps to generate host immune systems for pathogen elimination. The combinations of anti-adhesion process with antibiotics have shown excellent synergistic antibacterial activities [143]. For obvious reasons, researchers are attempting to use the OMV-NP combination to inhibit pathogen adhesion. Zhang et al. [19] coated PLGA NPs with OMVs (OM-NPs) from *Helicobacter pylori* (*H. pylori*) and demonstrated their binding to gastric epithelial cells (AGS cells). The treatment of AGS cells with OM-NPs caused reduced *H. pylori* adhesion, which is also dependent on the concentration and dosing sequence of the OM-NPs.

### 8.5. Drug Delivery

Biomimetic OMV-NPs have also been utilized as drug delivery vehicles. Gujrati et al. [20] used OMVs from *E. coli* to coat small interfering RNA (siRNA) to target kinesin spindle protein (KSP) for cancer cell killing. In this respect, a mutant *E. coli* strain with reduced endotoxicity toward human cells was used to generate OMVs. As a targeting ligand, the OMVs display a human epidermal growth factor receptor 2 (HER2)-specific affibody in the membrane. The overexpression of HER2, which is a transmembrane receptor with tyrosine kinase activity, is observed in 18–25% of breast cancers, ovarian cancers, gastric carcinoma, and salivary gland tumors. Therefore, HER2 is a promising target for both cancer diagnosis and therapy [20]. Hence, OMVs with an anti-HER2 affibody on the outer membrane surface were generated and therapeutic siRNA targeting KSP was loaded onto them. Injecting siRNA-packaged OMVs into an animal model resulted in targeted gene silencing with highly significant tumor growth regression, with no evidence of nonspecific side effects.

When it comes to the future of OMV-NPs for drug delivery application, one of the most pertaining issue that researchers deal with is the significant heterogeneity of OMVs. Therefore, it is critical to quantify data on OMV synthesis, drug loading, and culture conditions (isolation protocol, storage conditions, and others). Furthermore, different-sized OMVs from the same bacterial source should be evaluated for drug delivery application in order to understand the role of the OMV size parameter. Separation and purification techniques for drug-loaded OMV-NPs are another challenge that needs to be addressed. Depending on the properties of NPs in OMV-NPs, affinity-based separation or magnetic separation could be used in these cases. Therefore, these bioengineered OMV-NPs have remarkable potential for tackling biomedical challenges. Nonetheless, further research is required to transform this OMV-NP platform into a mainstay in biomedical applications.

## 9. Prospects and Challenges

The distinctive properties of cell membranes have enabled their use by researchers for various diverse biomedical applications utilizing the properties of NPs. In this review, the structure, preparation, and most importantly the applications of bacterial cell membranes or OMVs were thoroughly discussed. OMV-NPs have demonstrated immense potential for various biomedical applications such as photothermal therapy, immune modulation, and antibacterial activity. Despite the progress and the unlimited potential of OMV-NPs, there are at least four challenges that need to be addressed.

First, defined preparation methods for OMVs for target bacterial cells with extracting high quality and desired size need to be developed. For instance, it has been reported that OMVs derived from *H. pylori* ranging from 20 to 100 nm in size preferentially entered host cells via caveolin-mediated endocytosis, whereas larger OMVs ranging between 90 and 450 nm in size entered host epithelial cells via micropinocytosis and endocytosis [128]. This indicates that OMV-NPs with defined sizes are used differently in the bacterial regulation of virulence determinants and that the design and development of OMV-NP-based vaccines or therapeutics need to be refined. Until now, ultracentrifugation and precipitation methods for OMV preparation had difficulty overcoming size issues. Instead, column-based OMV preparation methods seemed to be promising. However, the components and biological activity should be pre-tested before their use in specific biomedical applications.

Second, alternative processes of OMV-NP preparation escaping multiple manual steps that can introduce process variability with high purity, integrity, and sidedness need to be investigated. Membrane extrusion and ultrasonic fusion were introduced as promising methods, but the two preparation processes have not been completely established for OMV-NP production. Specifically, it is not guaranteed to have high yield, loading capacity, or efficacy of NPs. Therefore, better user-friendly and impeccable processes requiring shorter times with high-efficacy OMV-NP preparation should be developed.

Third, the toxic nature of OMV-NPs should be addressed in further research work for an effective biomedical application. This could be overcome using engineered EVs with LPS-neutralizing peptides, which could reduce strong OMV-induced inflammatory responses compared with bacteria itself [144]. However, the challenges include developing universal-type-specific peptides instead of cell-type-specific ones.

Fourth, additional studies are needed to confirm the use of different arrays or inner cores for OMV-based biomedical applications, as very few NPs, such as PLGA, BSA, and Au NPs, are currently used despite the use of several NPs for other cell membranes. The scale-up of these biomimetic NPs is also an issue that must be adequately addressed.

Two other vital issues that need to be resolved regarding OMV coating of nanomaterials are whether cell membrane coating onto the core is successful and whether cell membrane coating orientation on nanomaterials is correct.

Notwithstanding these challenges, the OMV-based biomimetic NPs described in this review genuinely provide a unique opportunity to treat various health conditions, such as cancer and bacterial infections. Successfully addressing the above challenges will allow for the incorporation of a new class of OMV-NPs for cancer and infection theranostics to achieve personalized precision medicine.

## 10. Conclusions

In this review, the potential of OMV-coated NPs (OMV-NPs) for biomedical applications was discussed. OMV-NPs have synergistic biomedical applications and can enhance the activity of synthetic inner core NPs. These OMV-NP systems not only demonstrate their potential as therapeutic agents but also cause a paradigm shift in the development of new-generation materials for biomedical applications with NPs. Furthermore, the biomimetic properties of OMV-NPs could avoid the immune clearance used by single drugs and endows NPs with diverse cellular and functional mimicry. Encouragingly, the potential of these OMV-NP systems could contribute to their successful transfer to the clinical field, enabling the resolution of many critical issues in current management in the biomedical field and the development of optimized medicines to treat patients.
